# Elemental Profile and Health Risk of Fruška Gora Wines

**DOI:** 10.3390/foods12152848

**Published:** 2023-07-27

**Authors:** Ljilja Torović, Danijela Lukić, Tatjana Majkić, Ivana Beara

**Affiliations:** 1 Department of Pharmacy, Faculty of Medicine, University of Novi Sad, Hajduk Veljkova 3, 21000 Novi Sad, Serbia; 2 Center for Medical and Pharmaceutical Investigations and Quality Control, Faculty of Medicine, University of Novi Sad, Hajduk Veljkova 3, 21000 Novi Sad, Serbia; 3 Institute of Public Health of Vojvodina, Futoška 121, 21000 Novi Sad, Serbia; danijela.lukic@izjzv.org.rs; 4 Department of Chemistry, Faculty of Sciences, Biochemistry and Environmental Protection, University of Novi Sad, Trg Dositeja Obradovića 3, 21000 Novi Sad, Serbia; tatjana.majkic@dh.uns.ac.rs (T.M.); ivana.beara@dh.uns.ac.rs (I.B.)

**Keywords:** hazard index, lifetime cancer risk, margin of exposure, ICP-MS

## Abstract

The elemental composition of wine is influenced by endogenous sources and interventions from winemakers. The ICP-MS analysis of Fruška Gora wines (113) from vintages spanning across a decade (2011–2020), produced by 30 wineries and representing 18 autochthonous and international wine varieties, allowed a comprehensive insight into their elemental composition. Based on the mean concentrations of 23 investigated elements, B, Fe, and Mn, which were determined in mg per L of wine regardless of its colour or origin, were the most abundant. Red and white wines showed significant concentration differences in the case of B, Mn, and Sr (higher in red) as well as Be, Al, V, As, Mo, and Pb (higher in white). The elements of the highest toxicological concern were found in all (Pb and As) or almost all of the samples (Cd and Hg). Pb levels (maximum 47.1, 61.6, and 73.2 μg/L in red, rose, and white, respectively) were well below the legal limit. The applied risk assessment approaches (hazard quotient and index, margin of exposure) revealed no health concerns associated with consumption of Fruška Gora wines, except for a slightly increased lifetime cancer risk in the case of high wine consumption, and thus supported the promotion of Fruška Gora wines in the highly competitive international market.

## 1. Introduction

The elemental composition is an important determinant of wine quality and sensory attributes as well as health outcomes following its consumption. It is affected by various factors, such as vineyard soil, vinification process, and wine-making equipment. Based on their origin, the elements in wine could be considered endogenous or exogenous. The former (Ca, Mg, Fe, Zn, Na, K, and P) are more abundant and related to the grape variety and maturity as well as the type of vineyard soil (Mn, Ba, Ni, and other lithophile elements) and climate, whereas the latter (Al, Cr, Cu, Fe, Zn, Cd, and Pb) are introduced as impurities during the grape growth (environmental pollution, application of pesticides and fertilizers, etc.) and viticultural and winemaking processes [1,2]. Toxic elements as well as other chemical hazards in wine production could be associated with climate change, as corroborated by a study from Romania. The comparison of the elemental profiles of wines from the drought and post-drought years revealed a strong positive correlation between Pb and temperature, whereas the ground frost day frequency correlated with Pb and Cd [3].

The elemental profile represents a wine fingerprint and could be used in wine authentication [4,5], which is of utmost importance considering that wine is an important income commodity in primary wine-producing countries, such as Italy, France, Spain, USA, Argentina, and South Africa [6]. Therefore, despite analytical challenges, it is not surprising that an extremely large number of works on this topic have been published in the last decade [7], some of which further focus on disclosing the differences between organically and conventionally produced wines [8,9], which attract considerable attention nowadays. Additionally, some elements could contribute to the formation of undesirable flavor and aroma, and could thus cause deterioration of the organoleptic properties of the wine. Fe, Cu, Zn, and Cr can impact color attributes, browning, cloudiness, and astringency [2]. Fe, Al, and, most frequently, Cu can cause instabilities in wine. Cu instability is largely limited to white wine, and it is commonly observed as a fine haze and a mid-brown deposit that develops after bottling [10]. On the other hand, Cu, Fe, Mn, and Zn are used by yeasts for the activation of metallo-enzymes, and they thus exert a favorable influence on yeasts during alcoholic fermentation [7].

Research into element bioavailability in the soil-grapevine system could enable understanding of the element transportation chain. Research conducted in Oplenac wine growing area (Serbia) has pointed to Ba as the most bioavailable element in the soil-grapevine system, and has pointed to grape seeds and grape-vine leaves as better accumulators of Cu and Zn from the soil, respectively, compared to grape skin and pulp [11]. Results of a Romanian study investigating the transfer of toxic elements in the winemaking stages showed that Pb, Cu, and Cr concentrations decreased during the production of white wines, while in case of red wine varieties, only Pb and Cu concentrations were reduced [12]. Changes in the concentrations of the elements from soil to grape and wine have been studied in China: Mg, K, Ca, Cu, Zn, Rb, Sr, and Ba presented higher values of bioconcentration factor from soil to wine compared to Na, Fe, Mn, Al, Li, Cs, and Cd. The transfer from grape to wine was found to be greater than one for Na, Mg, K, Zn, Li, and Cs [13]. 

A study conducted in Spain, one of the best-known wine-producing countries worldwide, which has a long wine-making tradition and a famous wine-drinking culture, evaluated food safety performance in the protected denomination of origin wineries, applying the Hazard Analysis Critical Control Point (HACCP) system for identification, evaluation, and control of hazards. The worst performance level for the critical control points was for the control of toxic elements (Pb, Cd and As) and pesticide residues in grapes at harvest reception [14]. Thus, wine should be seen as a potential route of exposure to toxic elements, and this can lead to adverse health effects. Indeed, an association has been established between the following: low blood Pb concentration and chronic kidney disease and elevated systolic blood pressure [15]; prolonged and/or high exposure to Cd and renal dysfunction, leading to renal failure [16] as well as increased risk of lung, endometrium, bladder, and breast cancer [17]; chronic exposure to inorganic As and skin lesions as well as cancer of the urinary bladder, lung, and skin [17], developmental toxicity, neurotoxicity, cardiovascular diseases, abnormal glucose metabolism, and, lastly, diabetes [18]; and Hg and kidney (primarily), liver, nervous, immune, reproductive, and developmental system toxicity [19]. Therefore, wine quality and safety implications should precede any recommendation for wine consumption [20].

The significant growth of wine production in Serbia was supported by the national wine and viticulture development program, which was adopted in order to revive a long-lasting tradition dating from the Roman initiation of viticulture in third century [21]. In several years, the number of wineries increased by 58% and wine production increased by 30%, and exports reached 50% of the total production. One of the most important Serbian vineyard areas, situated on the foots of Fruška Gora, spreads over 2000 ha at 90–270 m of altitude, and it provides an excellent microclimate for growing grapes, influenced by its proximity to the Danube river [22]. In 2019, protected designation of origin Srem (wine area)—Fruška Gora (vineyard) was granted to 34 wineries with 147 wine labels.

The aims of this current study were to: (1) provide elemental profiles of wines produced in the Fruška Gora vineyard, (2) assess potential differences between elemental profiles of red and white wines as well as between wines originating from Fruška Gora and from the selected European countries, and (3) assess whether exposure to elements through wine consumption poses a health risk to the consumers, taking into account different consumption patterns and gender differences.

## 2. Materials and Methods

### 2.1. Sample Collection

The sample collection comprised a total of 131 bottled dry wines, with 113 (red, white, and rose) produced in 30 wineries on Fruška Gora and 18 (red) originating from several European countries, all of which were acquired in the city of Novi Sad (Serbia), which is situated on the foot of Fruška Gora in the Vojvodina wine region. Among Fruška Gora wines, 18 autochthonous and international wine varieties were represented with 91 monovarietal wines, while some additional varieties were represented in coupages. Information related to the grape variety, producer, vintage (spanning across the 2011–2020 decade), and alcoholic grade (from 11 to 15% (*v*/*v*)) was taken from the product labels. A description of the samples was presented in Table S1 (Supplementary Material). All of the samples were coded with a unique identification code, and they were stored unopened until analysis in an accredited analytical laboratory.

### 2.2. Chemicals

The standard solutions of individual elements were obtained from the following producers: As, Be, Mn, Pb, and V from AccuStandard Inc. (New Haven, CT, USA); Al, B, Ba, Cd, Co, Cr, Cu, Fe, Ni, Sb, Se, Sn, Sr, Te, Tl, and Zn from CPAchem (Stara Zagora, Bulgaria); Hg from Carlo Erba (Milan, Italy); and Mo from Chem Lab (Zedelgem, Belgium). Nitric acid (HNO_3_) 67–69% Normatom for trace metal analysis was obtained from VWR International (Leuven, Belgium). GenPure Water Purification System (Thermo Scientific, Thermoelectron LED, Langenselbold, Germany) was used for the production of ultrapure water for all analyses.

### 2.3. Sample Preparation

Based on the method MA-AS323-07:R2010 of the International Organization of Vine and Wine (OIV) [23] for multi-elemental analysis using ICP-MS, an aliquot of 1 mL of the sample was mixed with 0.5 mL of nitric acid, and ultrapure water was added up to 10 mL after 15 min. A dilution factor of 1:10 (*v*/*v*) was employed to eliminate matrix effects in ICP-MS analysis.

### 2.4. ICP-MS Analysis and Quality Assurance

The ICP-MS spectrometer 7700× (Agilent Technology, Waldbronn, Germany), equipped with an Ni sample and skimmer cones, a MicroMist nebulizer with a PEEK connector, a quartz spray chamber, and an integrated G3160B autosampler were used for analyses. The analysis was performed under the operational conditions previously described by Torović et al. (2022), as presented in Table S2. Either “No gas” mode or collision cell mode (He as collision gas, “He mode”) were used, and the latter was used when selectively attenuating polyatomic interferences was needed. The monitored and reported isotope(s), analysis mode and corresponding internal standard for each element are presented in Table S3, which also contains quantification and performance verification parameters. The analysis of calibration solutions prepared with the addition of nitric acid (1% in final solution) in a concentration range from 0.1 to 100 μg/L, and exceptionally for Hg from 0.01 to 10 μg/L, provided calibration curves with coefficients of determination (r^2^) greater than 0.998 for each of the analysed elements. The limit of detection (LOD) and quantification (LOQ) were determined based on standard deviation (SD) for reagent blanks measurements (*n* = 10; concentrations corresponding to 3-fold and 10-fold SD, respectively) (Table S3). Additionally, a recovery assay was conducted on red and white wine samples based on the difference between the concentration of elements in a natural sample (red and white wine, *n* = 5 replicates each) and a sample spiked with quality assurance (QC) solution of elements. The analysis of certified reference materials (CRMs 1643f and TM-DSW.3, chosen due to the similarity of their aqueous matrix to the prepared sample) and the analysis of proficiency testing (PT) samples (Table S4) enabled the method accuracy to be monitored.

### 2.5. Exposure and Risk Assessment—Input Data, Calculations and Interpretation Criteria

The exposure of adult men and women as well as of both sexes taken together was estimated based on data for the Population Average, Regular Drinkers Only and Chronic Heavy Drinkers regarding wine consumption in the Republic of Serbia, and this was taken from the latest WHO Global Status Report on Alcohol and Health [24] and presented in Table 1. The daily intake of elements was expressed on a body weight (bw) basis: men 82.0 kg, women 67.2 kg, and the average of both sexes was 73.9 kg [25].

Analytical results recorded as “non-detected” were substituted with ½ of the LOD of the analyzed element (middle bound (MB) approach) in order to make exposure and risk assessments more conservative.

The chronic exposure of adult men and women was determined by multiplying the concentration of each individual element (*i*) present in wine with the consumed volume of wine:

*EDI_i_* = *C_i_ × V/bw* (1)

where *EDI_i_* is estimated daily intake (mg/kg bw per day); *C_i_* is the concentration of element *i* (mg/L); *V* is the wine volume consumed (L per day); and *bw* is body weight (kg).

The hazard quotient (HQ), obtained as a ratio between chronic exposure and the oral reference dose of an element, represented a non-carcinogenic risk:

*HQ_i_* = *EDI_i_/RfD_i_* (2)

where *HQ_i_* is chronic hazard quotient, and where *RfD_i_* is reference dose (mg/kg bw per day). RfD values were obtained from the US EPA [26] (presented in Table S3).

The cumulative chronic risk (hazard index, HI) associated with a wine sample was calculated as a sum of HQs obtained for each individual element:

*HI* = Σ*HQ_i_* (3)

where: *HI*—hazard index; *HQ_i_*—hazard quotient. HQ and HI values lower than or equal to 1 indicated negligible risk, while higher values indicated increased risk. 

The margin of exposure (MOE) was obtained as a ratio between the BMDL and the exposure attributable to wine consumption:

*MOE_i_* = *BMDL_i_/EDI_i_* (4)

where *MOE_i_* is the margin of exposure (unitless), and where *BMDL_i_* is the benchmark dose lower confidence interval (mg/kg bw per day). BMDLs were taken from the EFSA: inorganic As (0.3–8) µg/kg bw/day [18], Pb 0.63 µg/kg bw/day for nephrotoxicity, and 1.5 µg/kg bw/day for cardiotoxicity [15]. Considering that BMDL values for both As and Pb are based on human data, adverse health effects are of low concern if exposure estimates are under the BMDL values (in case of As under the range of BMDLs), i.e., the obtained MOEs are higher than 1 (there is no need for an additional safety factor).

The tolerable weekly intake (TWI) was used as a supplemental risk indicator: exposure above the TWI was considered as a risk concern. TWIs for Cd of 2.5 µg/kg bw and for inorganic Hg of 4 µg/kg bw were obtained from the EFSA [16,19].

The assessment of lifetime cancer risk was based on the oral slope factor, presenting an estimate of the increased cancer risk corresponding to the oral dose of 1 mg/kg bw per day for a lifetime:

*LCR_i_* = *EDI_i_* × *q_i_* (5)

where *LCR_i_* is the lifetime cancer risk (unitless), and *q_i_* is the oral slope factor (kg bw per day/mg). The oral slope factor for As (1.5 kg bw per day/mg) was taken from the US EPA [26]. According to the WHO recommended guideline, 1 extra lifetime cancer case per 100,000 persons was considered maximum tolerable risk.

### 2.6. Statistical Analysis

The differences between the samples were evaluated using one-way analysis of variance (ANOVA) followed by comparison of the means by Tukey HSD test (*p* ≤ 0.05). The comparisons were conducted based on wine colour (red vs. white Fruška Gora wines), production year (2012 vs. 2014, i.e., extremely hot vs. extremely rainy year, according to the Republic Hydrometeorological Service of Serbia [27]), and origin (Fruška Gora red vs. imported red wines). The variability of the elemental profile among wine samples was assessed by using the principal component analysis (PCA). The data analyzed were standardized to account for the different magnitude, hence the responses and parameters contribute equally to the data set variance and to the principal component calculation. The classification was enhanced using partial least square discriminant analysis (PLS-DA) to generate variable importance scores of the potential element markers for wine samples. All statistical analyses were performed using the Microsoft Office Excel (v2019) and STATISTICA version 14.0.015. (TIBCO Software Inc., Palo Alto, CA, USA).

## 3. Results and Discussion

### 3.1. Elemental Profiles of Wines and Principal Component Analysis (PCA)

**Figure 1:**
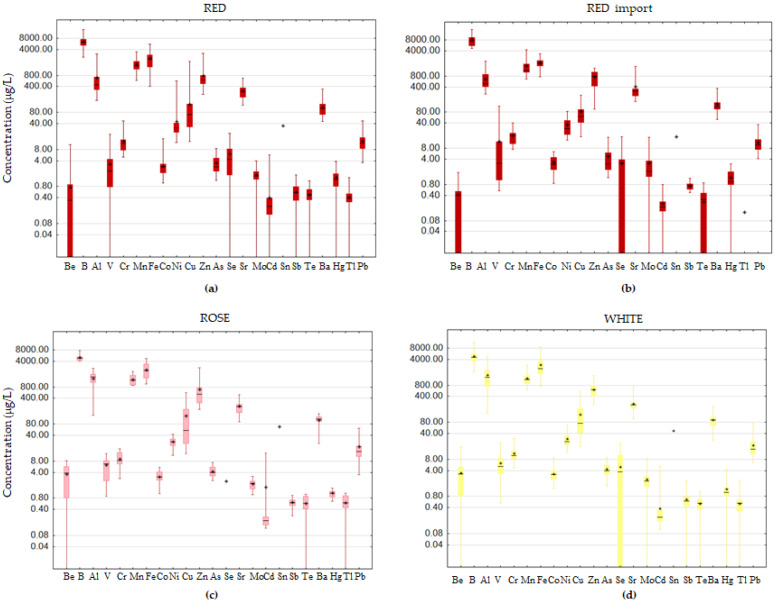
Box–Whisker plot of the elements’ concentrations: (**a**) red wine from Fruška Gora, (**b**) red wine (import), (**c**) rose wine from Fruška Gora, and (**d**) white wine from Fruška Gora (whiskers denoting min and max, □ interquartile range, −median, + mean).

Based on their mean concentrations, the most abundant elements in the wines were B, Fe, and Mn, determined in mg per L of wine regardless of its color or origin, followed by Zn (Figure 1, Table S5). Fe in wine reflects its content in grape and wine-making protocol, as long maceration (contact of grape skin and seeds with the must) leads to higher Fe levels, while variations in Mn concentration are a consequence of differences in soil composition. Zn is an element with a dual nature—due to its high mobility in soil, it is easily absorbed by grape (endogenous), while long contact of wine with stainless steel wine-making, handling, and storage equipment promotes its migration into wine (exogenous origin). Al was also abundant, especially in white (1477 μg/L) and rose wines (1374 μg/L). Along with natural Al sources, the bentonite agent used for wine fining could substantially contribute to the Al content in wine [2]. Cu showed a wide range of concentrations (13.1–1885 μg/L in red and 17.6–531 μg/L in white wines), which was not surprising considering its multiple ways of getting into wine, from accumulation in soil after the application of Cu-based fungicides (e.g., Bordeaux mixture) to Cu-containing equipment and/or Cu-salts used as additives for H_2_S removal. However, only one sample of white wine exceeded 500 μg/L, which was set as the approximate safe level in order to avoid Cu instability [10]. 

The elements of the highest toxicological concern, both those predominantly coming from anthropogenic sources (Pb and Cd) and those of geochemical origin (As), were found in all or almost all of the samples. Be, Co, Se, Mo, Sb, V, Tl, and Te were present in ultra-trace quantities (mean level below 10 μg/L), while Cr and Ni content, considered to be mainly related to wine interaction with stainless steel containers, went up to 48.0 and 572 μg/L, respectively. A relatively wide range of Sr content between 92.7 and 707 μg/L was obtained.

The statistical testing revealed significant concentration differences between red and white Fruška Gora wines in the case of B, Mn, and Sr (higher content in red), as well as Be, Al, V, As, Mo, and Pb (higher content in white). An increase in the content of elements in red wines could be a result of their more efficient extraction from grapes due to long maceration [1]. Higher As amounts in white wine compared with red were already reported for Serbian [28] as well as for some other wines (e.g., California wines [29]). The comparison of red wines based on origin, i.e., Fruška Gora vs. imported, revealed a significantly higher content of B, V, Cr, and Mo in imported red wines as opposed to Te and Tl content (Tables S5 and S6). In order to explore the possible influence of climate on the elemental composition of wine, wines from two production years with most contrasting climatic conditions were compared (Table S6). According to the Republic Hydrometeorological Service of Serbia, 2012 was extremely hot and 2014 was an extremely rainy year in the 2011–2020 decade [27]––specifically, the long-term deviation of temperature in 2012 was 2.4 °C, with 69 and 19 days when temperatures were above 30 and 35 °C, respectively, whereas 2014 had only 17 days with temperatures above 30 °C and not one above 35 °C, while the 70 rainy days resulted in a precipitation level of 698 mm (Table S7). However, comparison of the mean concentrations of each individual element revealed no substantial differences between red wines from 2012 and 2014 vintages, whereas in the case of white wines, significant differences were observed regarding Cr and Hg, with both showing higher concentrations in wines produced from the 2012 grape harvest. The difference related to the mean Cr concentration was retained even when all of the wines (red and white) were included in comparison between two years with distinguished climatic conditions (Figure S1).

**Figure 2:**
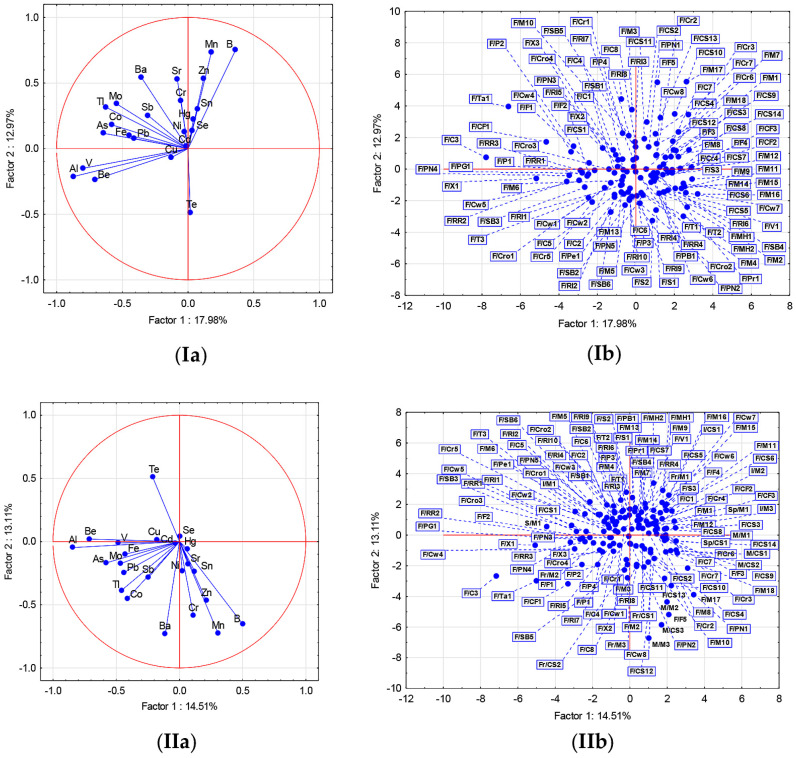
Principal component analysis of elemental content (**I**) in group of wines from Fruška Gora, (**II**) in groups of imported wines and wines from Fruška Gora: (**a**) Distribution of variables on loadings plot; (**b**) distribution of wine samples on scores plot. All samples were coded as locality/wine variety/sample no. Abbreviations: locality F—Fruška gora (Serbia), Fr—France, I—Italy, M—Macedonia, S—Slovenia, Sp—Spain. Wine variety: C1−8—Chardonnay; CF1−3—Cabernet Franc; Cr1,3,6—Cabernet Sauvignon, Merlot; Cr2—Merlot, Pinot Noir, Cabernet Sauvignon; Cr4—Skadarka, Cabernet Sauvignon; Cr5—Marselan, Merlot; Cr7—Probus, Marselan, Merlot; Cro1—Muscat Hamburg, Traminac, Cabernet Sauvignon; Cro2,4—Cabernet Sauvignon, Merlot; Cro3—Portugizer, Pinot Noir; CS1−14—Cabernet Sauvignon; Cw1—Italijanski rizling, Souvignon Blanc, Chardonnay, Župljanka; Cw2—Traminac, Muscat Otonel, Pinot Blanc, Pinot Gris; Cw3—Kevidinka, Chardonnay; Cw4,5,7—Sauvignon Blanc, Semillon; Cw6—Chardonnay, Bačka; Cw8—Chardonnay, Riesling; F1−5—Frankovka; M1−18—Merlot; MH1,2—Muskat Hamburg; P1−4—Portugizer; PB1—Pinot Blanc; Pe1—Petra; PG1—Pinot Grigio; PN1−5—Pinot Noir; Pr1—Probus; RI1−10—Rizling italijanski; RR1−4—Rajnski rizling; S1−3—Sila; SB1−6—Sauvignon Blanc; T1−3—Traminac; Ta1—Tamjanika; V1—Vranac X1—unspecified white wine; and X2,3—unspecified rose wine.

To elucidate whether the determined elements could be used to distinguish examined samples from the Fruška Gora region, the PCA was performed (Figure 2Ia,Ib). The first two components (F1 and F2) accounted for 30.95% of the explained variance (F1 17.98%, F2 12.97%; Table S8). General grouping of samples in the central part implies that examined samples have reasonably comparable elemental profiles. However, some exceptions could be noticed. For example, separation of Tamjanika wine (F/Ta1) was probably due to the highest content of Fe, Sr, and Tl. The uppermost content of Al contributed to the location of white wines F/C3 and F/X1. Interestingly, on the F/X1 sample product specification, grape variety was not listed, but F/C3 and F/X1 were produced in the same vinery, and the obtained result can imply that similarly significant amounts of bentonite, which could be a rich source of Al, were used during wine production. The position of red wine F/CF1 was mostly dependent on the highest Co concentration, while F/F5 and F/M17 were located in the right upper part mainly due to the distinguished content of B, Mn, and Zn.

**Figure 3:**
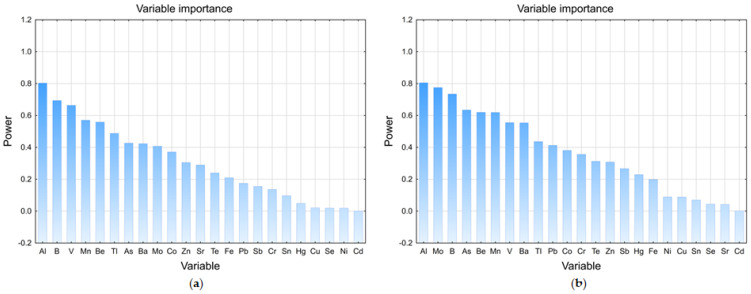
Variable importance plot (VIP) of the potential element markers for (**a**) wines from Fruška Gora; (**b**) imported and wines from Fruška Gora.

In order to evaluate the significance of elements, variable importance (VIP) was calculated by partial least square linear discriminant analysis (PLS-DA, Figure 3a). Since the discriminating power was lower than 1.0 for all of the elements, this implies that these elements could not be considered as candidate markers for the differentiation between the examined wines.

Further, PCA was performed to elucidate whether the determined elements could be used to distinguish the examined samples from the Fruška Gora region and the imported samples (Figure 2IIa,IIb). The first and second PCs (Factors 1 and 2) explained 14.51 and 13.11% of the total variance, respectively (Table S9). Again, the general grouping of the samples in the central part can be noticed, as well as the separation of F/C3, F/X1, F/Ta1, and F/CF1 in the lower left part. Certain groupings of some Fruška Gora and Macedonian samples in the right lower part (F/CS10, F/CS13, F/F5, F/M17, M/M3, M/M2, and M/CS3) were influenced by Mn and B content. Again, VIP showed variable power below 1.0 (Figure 3b). In conclusion, the assessment of the complete dataset revealed poor separation, and it points to the absence of any particular differences regarding the elemental profiles of the examined samples.

While comparisons between the studies should be taken cautiously, due to discrepancies between the investigated wine varieties, the capacity of the used analytical methods (the sensitivity and elements included in the analysis), the impact of outliers on the mean levels, etc., the importance of an interpretation of the results in view of the previous studies should still not be neglected. Regarding previous studies from a Central Balkan region, one conducted in Serbia included 63 wines but only one from the Fruška Gora vineyard [28]. Along with some similarities, a number of differences related to the Fruška Gora wines was observed regarding both the essential elements (the current study showed a two-fold higher mean Fe content in both red and white, a two-fold higher mean Mn in white, and a four-fold lower mean Cu in white wine) and toxic ones (from three-fold to five-fold lower Pb, Cd, and As content in both red and white). Several Croatian studies showed a substantial degree of similarity between the Fruška Gora and the continental Croatian wines: Cu and Zn mean levels were very similar in both red and white as well as Fe and Mn in red, while Pb was similar in white but three-fold lower in red wines (Cd and As not analysed) [10]; similar mean levels of all of the mentioned essential elements but a higher mean level of Pb, Cd, and As [30]; and similar ranges of all the mentioned essential and toxic elements [9]. This was not surprising given that the neighbouring Vojvodina region and continental Croatia, which are both parts of the Pannonian basin, share environmental conditions (soil, climate) to a much greater extent than Vojvodina and the remaining part of Serbia. An investigation comparing wines from Vojvodina and northern Croatia with wines from the Istria Adriatic region of Croatia found higher As content in the former, which was explained by As contamination of Pannonian basin groundwater [31]. This was corroborated with the very low As content observed in Italian wines originating from vineyards close to Istria [32]. The wines from the Romanian vineyards, also situated in the Pannonian basin, showed great similarity regarding mean Cu and Zn content, while in the case of Fe and Mn, two-fold lower mean concentrations were recorded. However, the overall Pb level was significantly higher (38.9 vs. 16.5 μg/L), while As was not analysed [33]. Another Romanian study recorded extremely high Cd content, up to 10.1 μg/L, as well as very high Pb levels (maximum 155 μg/L) in wines, which were attributed mainly to industrial pollution and road traffic [3]. Multi-element analysis of Macedonian wines emphasized the differences regarding the mean Fe (1890 vs. 2524 μg/L) and Cd contents (0.26 vs. 0.5 μg/L) relative to the Fruška Gora wines [34]. With respect to the European Union countries, data gathered by the EFSA included a relatively high number of toxic elements occurrence data in the wine samples. The Pb content was characterized with the mean and 95 percentile (P95) of 25 and 69 µg/kg, respectively. Similar to the current study, white wines showed higher mean Pb level than red ones (29 vs. 22 µg/kg) [35], but it should be noted that in the Fruška Gora wines, these levels were substantially lower (18.8 vs. 13.8 µg/kg). The mean As, Cd, and Hg levels recorded in the European database were comparable with the results obtained for the Fruška Gora wines to a variable extent: As 5.8 µg/kg [36] vs. 3.9 µg/kg; Cd 1.24 µg/kg [37] vs. 0.5 µg/kg; and Hg 0.4 µg/kg [19] vs. 1.3 µg/kg.

Finally, assessment of compliance of the investigated wine samples with the maximum limits recommended by the OIV [23] pointed out excessive levels of Cu and Cd in one red and one rose wine from Fruška Gora, respectively, as well as B content in two imported wines. Regarding Regulation 915/2023 [38], the limit established for Pb content in wine was 200 μg/L for wines produced from the 2001 fruit harvest to the 2015 fruit harvest and was 150 μg/L from 2016 to 2021, and these were not violated by any of the samples: the maximum determined levels in the Fruška Gora wines were 47.1 (red), 61.6 (rose), and 73.2 μg/L (white), and in the imported samples was 36.0 μg/L. These results were in agreement even with the more rigorous limit imposed on wines produced from the 2022 fruit harvest onwards (100 μg/L). The aforementioned study conducted on Serbian wines (only one from Fruška Gora) reported concentrations of toxic elements under the OIV limits [28].

### 3.2. Risk Assessment

Regarding the non-carcinogenic risk, not one of the investigated elements showed a HQ value over 1, revealing no risk for consumers regardless of the consumption scenario. Although elements found in wines do not resemble each other regarding consequent negative effects, i.e., do not exert their toxicity on the same organ and by the same mechanism, summation of risk quotients for each analysed element, resulting with HI, has been commonly used for the assessment of the cumulative toxicity of such mixtures. It should be noted that Pb and Te were exempted from the calculation, since oral reference doses for these elements have not been established [26].

**Figure 4:**
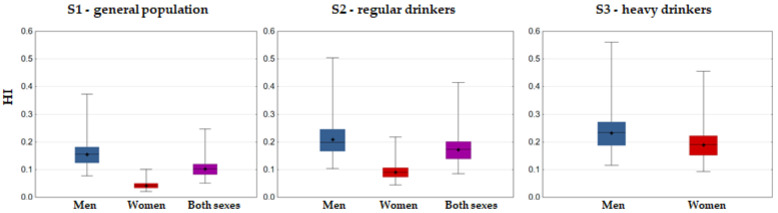
Box–Whisker plot of hazard index (HI) associated with Fruška Gora wine consumption (whiskers denoting min and max, □ interquartile range, − median, + mean).

**Figure 5:**
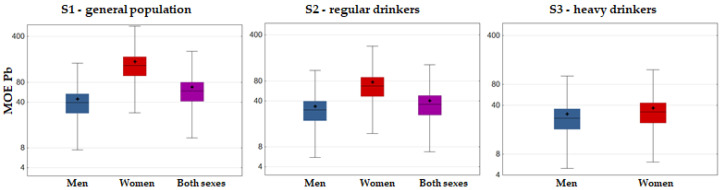
Box–Whisker plot of Pb MOE associated with Fruška Gora wine consumption (whiskers denoting min and max, □ interquartile range, − median, + mean).

**Figure 6:**
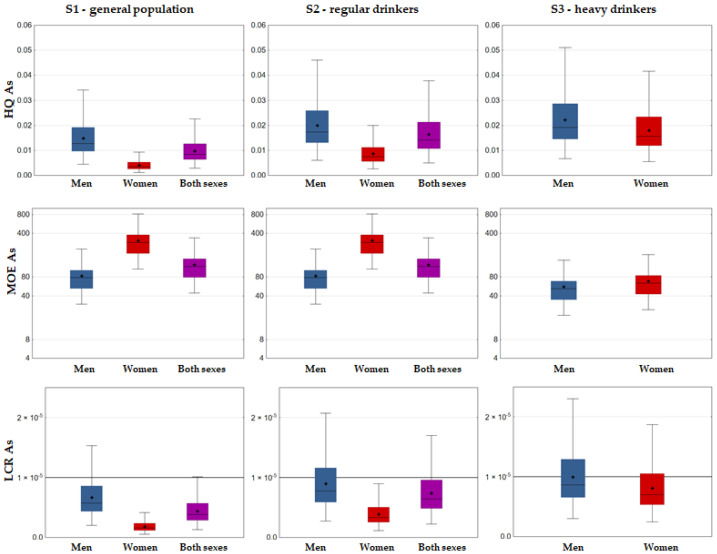
Box–Whisker plot of As risk metrics (HQ, MOE, LCR) associated with Fruška Gora wine consumption (whiskers denoting min and max, □ interquartile range, − median, + mean).

**Figure 7:**
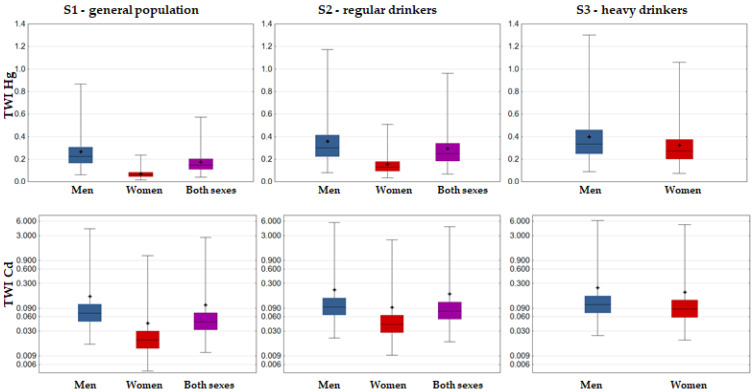
Box–Whisker plot of %TWI of Hg and Cd associated with Fruška Gora wine consumption (whiskers denoting min and max, □ interquartile range, − median, + mean).

The main risk assessment results are presented in Figure 4 (cumulative risk: HI approach), Figure 5 (Pb risk: MOE approach), Figure 6 (As risk: HQ, MOE and LCR approaches), and Figure 7 (Cd and Hg risk: TWI approach). 

Taking into account that HI reflects overestimated cumulative risk, results below 1 should be appraised as encouraging, and, indeed, even at high exposure (P95), HI values for both men and women were relatively low: 0.23 and 0.06 for population average, 0.31 and 0.13 for regular drinkers only, and 0.34 and 0.28 (at maximum extending up to 0.56 and 0.46) for heavy drinkers (Figure 4). It was interesting to note that the highest individual contribution to HI was from Tl (on average 34.7%), the element that was measured in very low concentrations (up to 2.2 µg/L) but with an extremely low RfD value (0.01 µg/kg bw/day). The second highest contribution was from B (on average 22.1%), driven by its high content in wines (mean 5648 µg/L) despite its moderate RfD (0.2 µg/kg bw/day). 

The risk related to Pb, estimated using the MOE approach, revealed no MOE below 1 in any consumption scenario (Figure 5). Although MOE associated with nephrotoxicity was lower than 10 for some individual wine samples (for 2, 4, and 6 samples across the consumption scenarios for males), both mean and P95 MOE values remained over 10 for both men and women. Thus, the risk from nephrotoxic as well as from the less sensitive cardiotoxic effects of Pb was unlikely.

Regarding As, the lowest HQ values were for more than an order of magnitude below 1, revealing no non-carcinogenic risk (Figure 6), and the carcinogenic risk evaluated via MOE approach also appeared negligible: both the mean and the high exposure estimates (P95) were below the range of BMDL_01_ (0.3–8 µg/kg bw/day) for at least an order of magnitude, i.e., MOE values were above 10 even when compared with the lower end of the BMDL_01_ range (Figure 6) and above 280 at the upper end in all of the consumption scenarios. However, lifetime cancer risk attributable to As exceeded the limit of tolerable risk of one extra lifetime cancer case per 100,000 persons (1 × 10^−5^) across the exposure scenarios at P87, P69, and P59 for males, whereas in the case of females, the breaking point was at P75 in a heavy wine consumption scenario (Figure 6). Importantly, analysis of the molecular mechanism and the epidemiological findings of As and alcohol toxicity postulated a hypothesis that they could exert combined toxic effects [39].

The risk of exposure to Cd and Hg, as evaluated against their respective TWIs, was in line with the HQ estimates: risk to wine consumers could be excluded, considering that both mean and high (P95) exposure levels were very low (<1% of TWI) for both men and women in every exposure scenario (Figure 7).

The comparison of risks associated with red wines from the Fruška Gora and red wines originating from several European countries, but available on the market of Vojvodina, reflected the findings obtained through the evaluation of the elements’ concentrations (results not shown), but, regardless of the differences, the risk indicators clearly demonstrated the safety of all of the investigated wines. The important exception was As LCR, which revealed risk concerns for a great majority of the samples (Figure 6).

Despite the multitude of studies investigating the multi-element profile of wines, those assessing health risk following exposure through wine consumption are less common. The majority of the published studies were based on the calculation of HQ and HI. It should be noted that, apart from different elements’ concentrations, differences in HQ values should be understood from the perspective of different national per capita wine consumption (the same is true for MOE and LCR approaches); in case of HI, additional influencing factors are selection and the number of analysed elements. In a previous Serbian study, estimated daily intake of toxic elements was well below respective toxicological thresholds, indicating no health risk [28]. A recent Croatian study showed that the average wine consumption does not expose consumers to significant levels of studied elements [10]. The HQ values for individual elements were significantly lower than 1 and followed the order Pb > Mn > Ni > Cu > Fe > Zn > Cr in red and Pb > Cu > Mn > Zn > Fe > Cr > Ni in white continental wines. Romanian wines also showed very low HQ and HI values (Fe, Cu, Mn, Ni, Cr, Pb), indicating an insignificant health risk for the exposed population [12]. An investigation conducted on wines from the Slovak market revealed that the type of wine and the alcohol content do not have a significant impact on the content of elements, while the age of wine was linked only with Zn concentration. The estimated dietary exposure was low, with the exception of Pb, which was the only element found to have exceeded the regulated maximum limit. Given the magnitude of the obtained MOE values, there was no risk at the mean Pb exposure level, but for the Slovak wines containing maximum Pb concentration, the possibility of adverse effects on the kidney and cardiovascular system in high and extreme consumption groups could not be excluded (MOE < 1) [40]. A review including 31 studies and 472 wine samples from 18 countries assessed the health risk of Pb ingestion [41]. The mean Pb content was 33.9 and 35.7 μg/L in red and white international wines (2.5- and two-fold higher compared to Fruška Gora wines), respectively. However, the consequent blood lead level in adult consumers was below the guidance value established by the US Center for Disease Control (5 μg/0.1 L). A Spanish study reported that Canarian drinkers are not exposed to unsafe levels of the eight elements studied, since maximum HI was 0.50. The main contributors to HI were B, accounting for 31–38% in South of Tenerife and La Palma wines, and Co, B, and Mn (28, 22, and 22%, respectively) in La Gomera wines [42]. In the Fruška Gora wines investigated in the current study, maximum HI for male regular drinkers was the same (but two-fold lower for women), and B showed the same contribution to HI as in La Gomera wines, while increments from Co and Mn were three-fold lower. In an Italian study, HQ values for Mn, Fe, Ni, Cu, Zn, and Sr were below 1 for at least an order of magnitude, thus demonstrating that there is no reason for any public health concern [43]. Several studies investigated As speciation in wines. The one conducted on wines from the Central European market showed the predominance of arsenite and arsenate, inorganic and more toxic forms of As, but estimated consumer exposure was low [44]. A screening level risk assessment of total inorganic As (the sum of As(III) and As(V)) conducted on Californian wines resulted in HQ values lower than 1, thus leading to insignificant non-carcinogenic risk. Importantly, the analysis showed that inorganic As was the primary form of As present in wine—total organic As content was ten- or more-fold lower. Based on these results and several previous studies reporting that inorganic As comprised approximately 70–100% of the total As in wines ([29], and the reference cited therein), it was concluded that, for risk assessment purposes, total As could be used as a conservative substitute for inorganic As [29].

The comparison between the exposure resulting from wine (alcoholic beverages) consumption and dietary background exposure could provide a useful context for the interpretation of the health risks attributed to wine. According to the assessment conducted on the EU level, the relative contribution of wine to the overall mean Pb chronic dietary exposure was 3.4% in adults and 4.5% in the elderly and the very elderly [35]. In the case of Cd, alcoholic beverages contributed with 0.9%, 20–36.9% of which was attributed to wine, depending on age (adults to the very elderly) [37]. The relative contribution of alcoholic beverages in adult population exposure across the EU countries ranged from 3–16% in the case of inorganic As (up to 90% of which attributed to beer) [36] and from 0.6 to 5.8% in case of inorganic Hg (no specific data for wine were available) [19]. The EFSA pointed out the lack of reliable data related to the content of inorganic As in alcoholic beverages among other less-studied foods, precluding more realistic dietary exposure estimates [45].

It is noteworthy that, besides risk, some studies investigated the potential benefits that could be attributed to various types of wine due to the fact that they contain essential elements. Thus, one study pointed out that a glass of Serbian wines could provide some amounts of essential elements, but not enough in a single dose to achieve the level recommended for labeling purposes (15% of recommended dietary allowance) [28]. The same was concluded for wines from the Slovak market [40] and from the Canary Islands [42], and this was part of the reason why potential benefits were not considered in the current study, along with the fact that wines contain a substantial amount of alcohol. It should be noted that the most recent official position of the health authorities is that no amount of alcohol consumption is without a risk to health [46].

### 3.3. Strengths and Limitations of the Study

Good representation of Fruška Gora wineries (30), wine varieties (18 varieties in monovarietal wines and additional in coupages), and wines (113) from vintages spanning across a decade (2011–2020) as well as wide coverage of elements (23) analyzed by a fit for purpose analytical method and internationally recognized risk assessment approaches have allowed a reliable insight into the elemental composition and health concerns associated with the consumption of Fruška Gora wines. Moreover, the presented risk estimates are another piece of the puzzle that will also contribute to the comprehensive understanding of the safety of Fruška Gora wines, along with the previously reported investigation of ochratoxin A, which revealed negligible health risk [47]. However, regarding the influence of climate on the presence of chemical hazards in grapes and wine, it should be noted that ochratoxin A content in Fruška Gora wines from 2014 vintage was significantly higher in comparison with wines from other production years due to the specific climatic conditions favorable for the colonization of grape berries by ochratoxigenic *Aspergillus* and *Pencillium* fungal species [47]. Along with safety, promising neuroprotective [48], anti-inflammatory, and antioxidant properties [20] of Fruška Gora wines provide a solid base for their promotion in the highly competitive international market and the popularization of regional wine tourism. 

With respect to the study’s limitations, rather low numbers of investigated imported red wines (18) limited the reliability of the comparison between them and the Fruška Gora wines in terms of both the elemental composition of the wines and the related risk estimates, which should be considered only as indication. Regarding analytical determination, it has to be noted that speciation of elements has not been performed, i.e., only total content of an element has been determined, which is of special importance in the case of As and Hg. However, it has been known that these elements occur in wines in their inorganic forms [18,19], and this has been taken into account in risk assessment. In order to overcome the uncertainty related to the consumed amount of wine, differences between the general adult population, regular drinkers, and chronic heavy drinkers were addressed through specific consumption scenarios, which included both men and women. 

## 4. Conclusions

The comprehensive elemental profiles of Fruška Gora wines could crucially contribute to the recognition of their geographical origin, and could provide oenologists with beneficial information relevant for the modulation of wine sensory properties and safety assurance. All of the risk indicators studied in the current research underlined the satisfactory safety profile of Fruška Gora wines in terms of their elemental composition, apart from the lifetime cancer risk of As exposure, which is related to certain consumption patterns that additionally lead to a substantial risk posed by a large intake of alcohol. Considering the plethora of factors possibly affecting the elemental composition of wine, generalization about the connections of each individual factor and the presence of elements in wine is extremely difficult to make. Although an exploration of the connections between the elemental profiles of wine and the vineyard soil type as well as the production conditions during grape growth and wine processing was out of the scope of the current study (mainly due to the lack of necessary input data), such research would be of a great interest in the future, since it could provide the oenologist with data of pivotal relevance for the improvement of wine safety. The research should not be limited to elemental contaminants, but should also include other particular hazards (e.g., pesticide residues) in order to identify not only potential hazards but also critical points for their control in the wine industry.

## Figures and Tables

**Table 1:** Wine consumption (L per day) by adult population (15+ years) in the Republic of Serbia [24].

Consumption Scenario	Population Average ^1^	Regular Drinkers Only ^2^	Chronic Heavy Drinkers ^3^
Men	0.094	0.127	0.141
Women	0.021	0.045	0.094
Both sexes	0.056	0.094	

^1^ Per capita consumption averaged across the entire adult population aged 15+. ^2^ Total adult population (15+) without abstainers. ^3^ For men and for women, 60 g and 40 g of alcohol per day, respectively. Percentage of wine in total recorded alcohol consumption: 42.5% [24].

## Data Availability

The datasets generated for this study are available on request to corresponding author.

## Supplementary Materials

The following supporting information can be downloaded at: https://www.mdpi.com/article/10.3390/foods12152848/s1, Table S1: Description of Fruška Gora wine samples; Table S2: ICP-MS operational parameters; Table S3: Quantification and performance verification parameters for ICP-MS determinations and oral reference doses of elements; Table S4: Results of proficiency testing of elements; Table S5: Profile of elements in wines (concentrations in µg/L); Table S6: One-way analysis of variance between element concentration in wine samples (*p*-value); Table S7: Weather conditions in the Republic of Serbia in 2012 and 2014; Table S8: Principal component analysis of elements in wines from Fruška Gora. Loadings of the variables for the first ten principal components, based on correlations; Table S9: Principal component analysis of elements in wines from Fruška Gora and imported wines. Loadings of the variables for the first ten principal components, based on correlations. Figure S1. Comparison of mean concentrations of elements in wines from production years 2012 and 2014 (extremely hot and extremely rainy year, respectively) (* *p* < 0.05).

## References

[1] Pohl P. (2007). What do metals tell us about wine?. Trends Anal. Chem..

[2] Tariba B. (2011). Metals in wine-impact on wine quality and health outcomes. Biol. Trace Elem. Res..

[3] Nechita C., Iordache A.M., Voica C., Costinel D., Botoran O.R., Popescu D.I., Suvar N.S. (2022). Evaluating the Chemical Hazards in Wine Production Associated with Climate Change. Foods.

[4] Teixeira R.J.S., Gomes S., Malheiro V., Pereira L., Fernandes J.R., Mendes-Ferreira A., Gomes M.E.P., Martins-Lopes P. (2021). A Multidisciplinary Fingerprinting Approach for Authenticity and Geographical Traceability of Portuguese Wines. Foods.

[5] Rapa M., Ferrante M., Rodushkin I., Paulukat C., Conti M.E. (2023). Venetian Protected Designation of origin wines traceability: Multi-elemental, isotopes and chemometric analysis. Food Chem..

[6] Fabjanowicz M., Plotka-Wasylka J. (2021). Metals and metal-binding ligands in wine: Analytical challenges in identification. Trends Food Sci. Technol..

[7] OIV (International Organisation of Vine and Wine) (2018). OIV Statistical Report on World Vitiviniculture. World Vitiviniculture Situation. http://www.oiv.int/public/medias/6371/oiv-statistical-report-on-world-vitiviniculture-2018.pdf.

[8] Drava G., Minganti V. (2019). Mineral composition of organic and conventional white wines from Italy. Heliyon.

[9] Vitali Čepo D., Pelajić M., Vinković Vrček I., Krivohlavek A., Žuntar I., Karoglan M. (2018). Differences in the levels of pesticides, metals, sulphites and ochratoxin A between organically and conventionally produced wines. Food Chem..

[10] Vitali Čepo D., Karoglan M., Borgese L., Depero L.E., Marguí E., Jablan J. (2022). Application of benchtop total-reflection X-ray fluorescence spectrometry and chemometrics in classification of origin and type of Croatian wines. Food Chem. X.

[11] Milićević T., Aničić Urošević M., Relić D., Vuković G., Škrivanj, Popović A. (2018). Bioavaillability of potentially toxic elements in soil-grapevine (leaf, skin, pulp and seed) system and environmental and health-risk assessment. Sci. Total Environ..

[12] Dumitriu G.D., Teodosiu C., Morosanu I., Plavan O., Gabur I., Cotea V.V. (2021). Heavy metals assessment in the major stages of winemaking: Chemometric analysis and impacts on human health and environment. J. Food Comp. Anal..

[13] Hao X., Gao F., Wu H., Song Y., Zhang L., Li H., Wang H. (2021). From Soil to Grape and Wine: Geographical Variations in Elemental Profiles in Different Chinese Regions. Foods.

[14] López-Santiago J., García García A.I., Gómez-Villarino M.T. (2022). An Evaluation of Food Safety Performance in Wineries. Foods.

[15] EFSA (European Food Safety Authority) (2010). Scientific opinion on lead in food. EFSA J..

[16] EFSA (European Food Safety Authority) (2009). Scientific opinion of the Panel on Contaminants in the Food Chain on a request from the European Commission on cadmium in food. EFSA J..

[17] IARC (International Agency for Research on Cancer) (2012). Arsenic, Metals, Fibers, and Dusts. IARC Monographs on the Evaluation of Carcinogenic Risks to Humans.

[18] EFSA (European Food Safety Authority) (2009). Scientific opinion on arsenic in food. EFSA J..

[19] EFSA (European Food Safety Authority) (2012). Scientific Opinion on the risk for public health related to the presence of mercury and methylmercury in food. EFSA J..

[20] Majkić T., Torović L., Lesjak M., Četojević-Simin D., Beara I. (2019). Activity profiling of Serbian and some other European Merlot wines in inflammation and oxidation processes. Food Res. Int..

[21] Ivanišević D., Jakšić D., Korać N. (2015). Vinogradarski Atlas.

[22] Vlastelica R. (2009). Serbian Wine Routes.

[23] OIV (International Organization of Vine and Wine) (2022). Multielemental analysis using ICP-MS, MA-AS323-07:R2010; Maximum acceptable limits of various substances contained in wine, OIV-MA-C1-01:R2019. Compendium of International Methods for Wine and Must Analysis.

[24] WHO (World Health Organization) (2018). Global Status Report on Alcohol and Health 2018.

[25] EFSA (European Food Safety Authority) (2012). Guidance on selected default values to be used by the EFSA Scientific Committee, Scientific Panels and Units in the absence of actual measured data. EFSA J..

[26] U.S. EPA (United States Environmental Protection Agency) (2020). Regional Screening Level (RSL) Summary Table.

[27] Republic Hydrometeorological Service of Serbia Agrometeorological Conditions in the Territory of the Republic of Serbia. https://www.hidmet.gov.rs.

[28] Đurđić S., Pantelić M., Trifković J., Vukojević V., Natić M., Tešić Ž., Mutić J. (2017). Elemental composition as a tool for the assessment of type, seasonal variability, and geographical origin of wine and its contribution to daily elemental intake. RSC Adv..

[29] Monnot A., Tvermoes B., Gerads R., Gürleyük H., Paustenbach D. (2016). Risks associated with arsenic exposure resulting from the consumption of California wines sold in the United States. Food Chem..

[30] Leder R., Petric I.V., Jusup J., Banović M. (2021). Geographical Discrimination of Croatian Wines by Stable Isotope Ratios and Multielemental Composition Analysis. Front. Nutr..

[31] Fiket Ž., Mikac N., Kniewald G. (2011). Arsenic and other trace elements in wines of eastern Croatia. Food Chem..

[32] Fermo P., Comite V., Sredojević M., Ćirić I., Gašić U., Mutić J., Baošić R., Tešić Ž. (2021). Elemental Analysis and Phenolic Profiles of Selected Italian Wines. Foods.

[33] Geana E.I., Marinescu A., Iordache A.M., Sandru M., Ionete R.E., Bala C. (2014). Differentiation of Romanian Wines on Geographical Origin and Wine Variety by Elemental Composition and Phenolic Components. Food Anal. Methods.

[34] Ivanova-Petropulos V., Wiltsche H., Stafilov T., Stefova M., Motter H., Lankmayr E. (2013). Multielement analysis of Macedonian wines by inductively coupled plasma–mass spectrometry (ICP-MS) and inductively coupled plasma–optical emission spectrometry (ICP-OES) for their classification. Maced. J. Chem. Chem. Eng..

[35] EFSA (European Food Safety Authority) (2012). Lead dietary exposure in the European population. EFSA J..

[36] EFSA (European Food Safety Authority) (2014). Dietary exposure to inorganic arsenic in the European population. EFSA J..

[37] EFSA (European Food Safety Authority) (2012). Cadmium dietary exposure in the European population. EFSA J..

[38] EC (European Commission) (2023). Commission Regulation (EU) 2023/915 of 25 April 2023 on maximum levels for certain contaminants in food and repealing Regulation (EC) No 1881/2006. Off. J. EU.

[39] Bao L., Shi H. (2010). Potential molecular mechanisms for combined toxicity of arsenic and alcohol. J. Inorg. Biochem..

[40] Semla M., Schwarcz P., Mezey J., Binkowski L., Błaszczyk M., Formicki G., Gren A., Stawarz R., Massanyi P. (2018). Biogenic and risk elements in wines from the Slovak market with the estimation of consumer exposure. Biol. Trace Elem. Res..

[41] Towle K.M., Garnick L.C., Monnot A.D. (2017). A human health risk assessment of lead (Pb) ingestion among adult wine Consumers. Int. J. Food Contam..

[42] Gutiérrez A., Rubio C., Moreno I., González A., Gonzalez-Weller D., Bencharki N., Hardisson A., Revert C. (2017). Estimation of dietary intake and target hazard quotients for metals by consumption of wines from the Canary Islands. Food Chem. Toxicol..

[43] Dalipi R., Borgese L., Zacco A., Tsuji K., Sangiorgi E., Piro R., Bontempi E., Depero L.E. (2015). Determination of trace elements in Italian wines by means of total reflection X-ray fluorescence spectroscopy. Int. J. Environ. Anal. Chem..

[44] Huang J.-H., Hu K.-N., Ilgen J., Ilgen G. (2012). Occurrence and stability of inorganic and organic arsenic species in wines, rice wines and beers from Central European market. Food Addit. Contam..

[45] EFSA (European Food Safety Authority) (2021). Scientific report on the chronic dietary exposure to inorganic arsenic. EFSA J..

[46] Burton R., Sheron N. (2018). No level of alcohol consumption improves health. Lancet.

[47] Torović L., Lakatoš I., Majkić T., Beara I. (2020). Risk to public health related to the presence of ochratoxin A in wines from Fruska Gora. LWT–Food Sci. Technol..

[48] Beara I., Torović L., Pintać D., Majkić T., Orčić D., Mimica-Dukić N., Lesjak M. (2018). Polyphenolic profile, antioxidant and neuroprotective potency of grape juices and wines from Fruška Gora region (Serbia). Int. J. Food Prop..

