# Supplementary material for: Elemental Profile and Health Risk of Fruška Gora Wines
Source: Foods. 2023 Jul 27;12(15):2848. doi: 10.3390/foods12152848 (PMC10417728; doi:10.3390/foods12152848)
Supplement: Supplementary file 1 [file foods-12-02848-s001.zip › foods-2498362-supplementary.pdf]

Supplementary material

Elemental profile and health risk of wines from Fruška Gora

The supplementary material includes:

Content		Page
Table S1	Description of Fruška Gora wine samples	2
Table S2	ICP-MS operational parameters	3
Table S3	Quantification and performance verification parameters for ICP-MS determinations and oral reference doses of elements	4
Table S4	Results of proficiency testing of elements by ICP-MS	5
Table S5	Profile of elements in wines (concentrations in μg/L)	6
Table S6	One-way analysis of variance between element concentration in wine samples (p-value)	7
Table S7	Weather conditions in the Republic of Serbia in 2012 and 2014	7
Table S8	Principal component analysis of elements in wines from Fruška Gora. Loadings of the variables for the first ten principal components, based on correlations	8
Table S9	Principal component analysis of elements in wines from Fruška Gora and imported wines. Loadings of the variables for the first ten principal components, based on correlations	9
Figure S1	Comparison of mean concentrations of elements in wines from production years 2012 and 2014 (extremely hot and extremely rainy year, respectively)	10

Table S1. Description of Fruška Gora wine samples.

Sample code	Wine variety	Winery
**C1–8**	*Chardonnay*	Kovačević, Mačkov podrum, Vinum, Dulka, Belo brdo, Šijački, Došen, Petrović
**CF1–3**	*Cabernet Franc*	Đurđić, Urošević
**Cr1,3,6**	*Cabernet Sauvignon, Merlot*	Kovačević, Mačkov podrum
**Cr2**	*Merlot, Pinot Noir, Cabernet Sauvignon*	Orfelin
**Cr4**	*Skadarka, Cabernet Sauvignon*	Faculty of Agriculture Novi Sad
**Cr5**	*Marselan, Merlot*	Bjelica
**Cr7**	*Probus, Marselan, Merlot*	Deurić
**Cro1**	*Muscat Hamburg, Traminac, Cabernet Sauvignon*	Orfelin
**Cro2**	*Cabernet Sauvignon, Merlot*	Kovačević
**Cro3**	*Portugizer, Pinot Noir*	Mačkov podrum
**Cro4**	*Merlot, Cabernet Sauvignon*	Došen
**CS1–14**	*Cabernet Sauvignon*	Dulka, Petrović, Kosović, Bajilo, Mrđanin, Šijački, Trivanović, Živanović, Adžić, Do kraja sveta
**Cw1**	*Italijanski rizling, Souvignon Blanc, Chardonnay, Župljanka*	Orfelin
**Cw2**	*Traminac, Muscat Otonel, Pinot Blanc, Pinot Gris*	Kovačević
**Cw3**	*Kevidinka, Chardonnay*	Faculty of Agriculture Novi Sad
**Cw4,5,7**	*Sauvignon Blanc, Semillon*	Bjelica, Vinum
**Cw6**	*Chardonnay, Bačka*	Vindulo
**Cw8**	*Chardonnay, Riesling*	Patrijaršijsko
**F1–5**	*Frankovka*	Vindulo, Trivanović, Podrum Probus
**M1–18**	*Merlot*	Šijački, Mačkov podrum, Dulka, Kiš, Šukac, Došen, Kosović, Podrum Probus, Međanin, Živanović, Vinoprodukt, DBDM DiBonis, Deurić, Patrijaršijsko, Veranda Bećar, Petrović, Adžić
**MH1,2**	*Muskat Hamburg*	Bajilo, Žabić
**P1–4**	*Portugizer*	Mačkov podrum, Bajilo
**PB1**	*Pinot Blanc*	Podrum Probus
**Pe1**	*Petra*	Faculty of Agriculture Novi Sad
**PG1**	*Pinot Grigio*	Trivanović
**PN1–5**	*Pinot Noir*	Dumo, Mačkov podrum, Vinum, Belo brdo
**Pr1**	*Probus*	Živanović
**RI1–10**	*Rizling italijanski*	Faculty of Agriculture Novi Sad, Šijački, Bajilo, Kosović
**RR1–4**	*Rajnski rizling*	Šijački, Mačkov podrum, Kiš
**S1–3**	*Sila*	Bajilo, Žabić, Petrović
**SB1–6**	*Sauvignon Blanc*	Đurđić, Mačkov podrum, Dulka, Belo brdo, Šukac, Kovačević
**T1–3**	*Traminac*	Đurđić, Mačkov podrum, MCC
**Ta1**	*Tamjanika*	Živanović
**V1**	*Vranac*	Urošević
**X1**	*unspecified white wine*	Vinum
**X2,3**	*unspecified rose wine*	Dulka

Table S2. ICP-MS operational parameters

Parameter	Analysis mode	
	**No Gas mode**	**He mode**
RF- power (W)	1550	1550
Sample depth (mm)	8	8
Nebulizer pump speed (rps)	0.1	0.1
Plazma gas flow (L/min)	15.0	15.0
Carrier gas flow (L/min)	1.2	1.2
Dilution gas flow (L/min)	1.0	1.0
Aux gas (L/min)	0.90	0.90
Spray chamber	Water cooled double pass	
Lens voltage	4.5	4.5
Mass resolution	0.8	0.8
Integration time points/ms	3	3
Points per peak	3	3
Replicates	3	3

Tuning solution: cerium (Ce), cobalt (Co), lithium (Li), magnesium (Mg), thallium (Tl), and ittrium (Y) (10 µg/mL each, in 2% v/v HNO_3) (Agilent Technologies, Waldbronn, Germany).

Prior to the analysis, the ICP-MS system was equilibrated for 30 min and then checked in terms of sensitivity, stability and performance, using the diluted tune solution (1 µg/L of each element), whereas auto-tune and calibration mass tests were performed when it was necessary for optimisation of the instrument.

Internal standard: the mixture of bismut (Bi), germanium (Ge), indium (In), lithium (Li), lutetium (Lu), rhodium (Rh), scandium (Sc), and terbium (Tb), (10 µg/mL each, in 10% v/v HNO_3), provided by Agilent Technologies (Waldbronn, Germany). Internal standard was added at a constant rate and concentration to all calibration standards and unknown samples.

Table S3. Quantification and performance verification parameters for ICP-MS determinations and oral reference doses of elements

Element	Symbol	Monitored isotopes	Reported isotope	Internal standard (isotope)	Analysis mode	LOD (µg/L)	LOQ (µg/L)	Recovery[a,b] (%)		RfD (mg/kg bw/day)
								red	white	
Beryllium	**Be**	9	9	Sc (45)	No gas	0.3	1	93.7	86.0	0.003
Boron	**B**	11	11	Sc (45)	No gas	560	1800	89.1	83.0	0.2
Aluminium	**Al**	27	27	Sc (45)	He	4.6	13.8	104.2	98.2	1
Vanadium	**V**	51	51	Sc (45)	He	0.3	1	100.7	111.9	0.005
Chromium	**Cr**	50, 52	52	Sc (45)	He	0.5	1.6	85.3	86.5	1.5
Manganese	**Mn**	55	55	Sc (45)	He	0.5	1.4	107.7	106.8	0.14
Iron	**Fe**	54, 56	56	Sc (45)	He	4.7	14	94.5	94.0	0.7
Cobalt	**Co**	59	59	Sc (45)	He	0.3	1	90.1	95.4	0.0003
Nickel	**Ni**	60, 61	60	Sc (45)	He	0.3	1	95.1	96.3	0.02
Copper	**Cu**	63, 65	65	Sc (45)	He	0.5	1.6	90.4	91.3	0.04
Zinc	**Zn**	66, 67, 68	66	Ge (74)	He	1.1	3.3	90.0	102.1	0.3
Arsenic	**As**	75	75	Ge (74)	He	0.3	1	111.2	159.3	0.0003[c]
Selenium	**Se**	76, 77, 78	78	Ge (74)	He	2.5	7.5	119.6	173.1	0.005
Strontium	**Sr**	88	88	Ge (74)	He	0.5	1.5	87.1	89.2	0.6
Molybdenum	**Mo**	95	95	Ge (74)	He	1	3	101.7	114.6	0.005
Cadmium	**Cd**	111, 114	111	In (115)	He	0.1	0.3	108.3	92.7	0.0005
Tin	**Sn**	117, 118	117	In (115)	He	30	90	50.8	58.8	0.6
Antimony	**Sb**	121, 123	121	In (115)	No gas	0.3	1	75.4	115.6	0.0004
Tellurium	**Te**	125, 126	125	In (115)	No gas	0.3	1	125.2	118.1	-
Barium	**Ba**	135	135	In (115)	No gas	2	6	83.0	80.8	0.2
Mercury	**Hg**	200, 201, 202	average	Bi (209)	No gas	0.6	1.8	100.7	86.7	0.0003[c]
Thallium	**Tl**	203, 205	205	Bi (209)	No gas	0.3	1	83.6	83.2	0.00001
Lead	**Pb**	206, 207, 208	average	Bi (209)	No gas	0.3	1	95.9	104.9	-

[a] Recovery assay: recovery was determined based on the difference between concentration of elements in natural sample (red and white wine, n=5 replicates each) and sample spiked with QC standard of 23 elements (red and white wine, n=5 replicates) prepared from the individual standards of Be, Te and Tl obtained from Carl Roth + Co. KG (Karlsruhe, Germany), B, Al, V, Ni, Cu, Zn, Se, Sb and Ba from AccuStandard Inc (New Haven, CT, USA), Cr, Fe, Cd, and Sn from Merck (Darmstadt, Germany), Co and Sr from Carlo Erba (Milan, Italy), Mn, As, Mo, and Pb from CPAchem (Stara Zagora, Bulgaria), Hg from Carlo Erba (Milano, Italy).
[b] Recovery assay spike levels: 1 µg/L Be, Co, Mo, Cd, Te, Hg, Tl; 10 µg/L V, Ni, As, Se, Sb, Pb; 100 µg/L Cr, Mn, Cu,; 200 µg/L Zn, Sr; 500 µg/L Al, Fe, Sn, Ba; 10000 µg/L B.
[c] inorganic form.

Table S4. Results of proficiency testing of elements.

PT provider:	IFA				LGC		ERA
Year:	2020		2021		2021	2022	2023
Round:	M154A	M154B	M159A	M159B	AQ599	AQ621	Q500
Element	z-score[a]						
**Al**	-0.86	0.08	-0.28	0.62	-0.64	-	-0.45
**As**	-0.52	0.54	0.25	0.50	0	-1.32	-1.72
**Pb**	-0.54	-0.26	-0.82	-0.92	-1.18	-0.73	-1.37
**Cd**	-2.89	1.17	-0.39	0.15	0.71	-1.44	-1.71
**Cr**	-1.30	0.12	0.7	0.72	-0.32	-0.85	-1.63
**Fe**	-1.05	0.17	0.13	1.06	0.84	-	0.07
**Cu**	-0.62	0.10	-0.18	0.15	0.27	-	-0.51
**Mn**	-1.12	0.50	-0.32	0.64	-0.59	-	-1.60
**Ni**	-0.96	0.78	0.61	1.13	0	-0.68	-1.50
**Zn**	-1.44	-0.36	-1.17	-0.54	0.46	-	-0.16
**Se**	-0.62	-0.60	-	2.34	0.49	-1.60	0.32
**Hg**	-	-	0.38	0.48	0.47	-1.48	-
**Ba**	-	-	-	-	-0.13	-	-0.48
**Be**	-	-	-	-	-0.14	0.42	-0.62
**Co**	-	-	-	-	-0.32	-0.43	-0.24
**Mo**	-	-	-	-	0.84	-1.26	-2.29
**Sn**	-	-	-	-	-0.2	-1.05	-
**Sb**	-	-	-	-	-	-1.29	-0.45
**V**	-	-	-	-	-0.15	-1.02	-0.13
**B**	-	-	-	-	-	-	0.00
**Sr**	-	-	-	-	-	-	-1.28
**Tl**	-	-	-	-	-	-	-1.96

IFA – Sample Metals. Reports: IFA Proficiency testing scheme for water analysis. University of Natural Resources and Life Sciences, Vienna. Department of Agrobiotechnology IFA-Tulln, Tulln Austria. Round 149 Metals, 2019; Round 154 Metals, 2020; Round 159 Metals, 2021.

LGC - Sample 17C - Metals (preserved in 0.5% Nitric acid). Report: LGC Proficiency testing. Water Chemistry (Aquacheck). 1 Chamberhall Business Park, Chamberhall Green, Bury, United Kingdom. Round 599, 2021.

ERA A Waters Company. Report: WP Trace Metals, Q500. 16341 Table Mountain Parkway, Golden, Co, USA. 2023.

[a]z-score evaluation criterion: scores between -2 and 2 are considered satisfactory.

Table S5. Profile of elements in wines (concentrations in μg/L).

Element	Fruška gora wines								Imported wines	
	all		red		white		rose		red	
	min-max	mean±SD	min-max	mean±SD	min-max	mean±SD	min-max	mean±SD	min-max	mean±SD
**Be**[rw]	n.d. -17.4	2.1±2.9	n.d.-10.7	0.8±1.6	n.d.-17.4	3.4±3.4	n.d.-8.4	3.5±2.8	n.d.-1.7	0.4±0.4
**B**[rw, rr]	1834-13608	5648±2213	2494-13608	6511±2444	1834-11613	4776±1690	3902-7717	5061±1180	4562-15665	8210±3107
**Al**[rw]	136-4671	1085±849	167-3018	693±605	139-4671	1477±930	136-2524	1374±676	252-2048	656±427
**V**[rw, rr]	n.d.-22.7	4.8±4.2	n.d.-20.1	3.3±3.7	0.5-22.7	6.3±4.2	0.9-12.8	6.3±3.8	0.5-118	12.6±27.4
**Cr**[rr]	2.8-48.0	12.2±6.8	5.0-48.0	13.3±8.1	4.6-29.4	11.7±5.4	2.8-16.9	9.0±4.1	7.5-40.9	18.2±8.5
**Mn**[rw]	588-3510	1405±544	588-3510	1596±603	595-2711	1212±420	867-2074	1270±380	664-4239	1548±933
**Fe**	408-8584	2524±1336	408-5488	2329±1135	741-8584	2799±1539	956-4690	2329±1231	773-3336	1867±587
**Co**	1.0-15.4	3.0±1.8	1.0-15.4	2.9±2.1	1.3-8.7	3.2±1.4	1.1-5.5	3.0±1.3	0.9-6.4	3.3±1.6
**Ni**	11.6-572	36.5±54.2	12.2-572	45.3±76.0	12.9-69	28.4±12.7	11.6-43.1	27.0±9.2	13.6-85.5	35.8±21.3
**Cu**	12.7-1885	132±208	13.1-1885	133±265	17.6-531	131±126	12.7-556	134±180	17.0-238	84.9±62.8
**Zn**	200-3085	705±508	247-3085	786±622	246-1461	613±238	200-2647	698±683	96-1335	758±363
**As**[rw]	1.2-8.9	3.9±1.8	1.2-8.6	3.4±1.9	1.5-8.9	4.3±1.7	2.4-7.5	4.2±1.5	1.2-16.0	4.8±4.1
**Se**	n.d.-22.0	5.2±6.1	n.d.-22.0	6.1±6.4	n.d.-22.0	4.9±6.0	n.d.-11.3	2.3±3.7	n.d.-16.9	3.1±3.9
**Sr**[rw]	92.7-707	281±123	123.6-669	312±134	99.0-707	255±105	92.7-487	242±104	157-1465	402±320
**Mo**[rw, rr]	n.d.-8.6	2.0±1.1	n.d.-4.0	1.7±0.7	n.d.-8.6	2.3±1.4	1.0-3.2	1.9±0.6	n.d.-16.1	3.1±3.6
**Cd**	n.d.-13.0	0.5±1.4	n.d.-5.7	0.4±0.8	0.1-5.3	0.4±0.8	0.12-13.0	1.6±3.9	n.d.-0.8	0.2±0.2
**Sn**	n.d.-915	43.5±127	n.d.-384	35.1±69.2	n.d.-915	47.6±157	n.d.-678	67.8±203	n.d.-52.3	16.7±21.8
**Sb**	n.d.-2.1	0.6±0.3	n.d.-1.7	0.6±0.4	n.d.-2.1	0.7±0.4	0.3-1.0	0.6±0.2	0.5-1.2	0.7±0.2
**Te**[rr]	n.d.-1.1	0.5±0.3	n.d.-1.1	0.5±0.3	n.d.-1.1	0.5±0.3	n.d.-1.0	0.6±0.3	n.d.-0.9	0.3±0.3
**Ba**	24.4-340	102±46.1	45.0-340	109±50.7	25.7-214	94±42.0	24.4-150	101±36.1	51.4-373	136±79.5
**Hg**	n.d.-4.3	1.3±0.8	n.d.-3.8	1.4±0.8	n.d.-4.3	1.3±0.8	0.7-1.5	1.1±0.2	n.d.-3.0	1.2±0.7
**Tl**[rr]	n.d.-2.2	0.5±0.4	n.d.-1.4	0.4±0.3	n.d.-2.2	0.5±0.4	n.d.-1.1	0.6±0.4	n.d.-0.8	0.1±0.3
**Pb**[rw]	3.5-73.2	16.5±10.7	3.6-47.1	13.8±7.7	6.5-73.2	18.8±11.5	3.5-61.6	20.0±16.8	4.2-36.0	11.7±7.3

SD – standard deviation

rw - elements with statistically different concentrations in Fruška Gora red and white wines

rr- elements with statistically different concentrations in Fruška Gora and imported red wines

Table S6. One-way analysis of variance between element concentrations in wine samples (p-value)

Element	Wine samples				
	F *red* and F white (p-value)	F *red* and I *red* (p-value)	Red 2012 and 2014 (p-value)	White 2012 and 2014 (p-value)	All 2012 and 2014 (p-value)
**Be**	1.02×10^{-6}*	0.3635	0.5112	0.2618	0.1586
**B**	8.44×10^{-5}*	0.0195*	0.4636	0.5914	0.7571
**Al**	1.56×10^{-6}*	0.8095	0.3272	0.5990	0.5908
**V**	0.0002*	0.0150*	0.8248	0.8376	0.8371
**Cr**	0.2570	0.0005*	0.5180	0.0367*	0.0206*
**Mn**	0.0004*	0.7996	0.3052	0.4495	0.4183
**Fe**	0.0795	0.1035	0.9382	0.7780	0.6786
**Co**	0.2867	0.3951	0.2059	0.2037	0.0740
**Ni**	0.1340	0.6022	0.2977	0.7797	0.1345
**Cu**	0.9722	0.4542	0.2003	0.5500	0.1200
**Zn**	0.0757	0.8609	0.5528	0.2151	0.5012
**As**	0.0099*	0.0573	0.6947	0.1812	0.2040
**Se**	0.3418	0.0643	0.2255	0.9004	0.2785
**Sr**	0.0208*	0.0930	0.7577	0.8299	0.4814
**Mo**	0.0022*	0.0047*	0.2373	0.8798	0.4603
**Cd**	0.8602	0.3715	0.3935	0.8919	0.4403
**Sn**	0.5956	0.2722	0.2349	0.9955	0.4837
**Sb**	0.2855	0.1100	0.1592	0.3935	0.0886
**Te**	0.7207	0.0067*	0.5605	0.2415	0.7206
**Ba**	0.1112	0.0892	0.3149	0.6239	0.2440
**Hg**	0.3815	0.3577	0.2503	0.0261*	0.2622
**Tl**	0.1813	0.0014*	0.6819	0.7866	0.6700
**Pb**	0.0095*	0.3238	0.6358	0.4781	0.2710

* statistically different, $p \leq 0.05$

F- Fruška gora wines

I- imported wines

Table S7. Weather conditions in the Republic of Serbia in 2012 and 2014 [27].

Year	Long-term deviation of temperature (°C)	No. of days with T > 20°C	No. of days with T > 30°C	No. of days with T > 35°C	No. of rainy days	Precipitation amount (mm)
**2012**	2.4	150	68	19	39	279
**2014**	0.7	136	17	0	70	698

Table S8. Principal component analysis of elements in wines from Fruška Gora. Loadings of the variables for the first ten principal components, based on correlations

	Factor 1	Factor 2	Factor 3	Factor 4	Factor 5	Factor 6	Factor 7	Factor 8	Factor 9	Factor 10
**Be**	-0.7097	-0.2360	0.0657	-0.4104	0.1745	-0.0016	0.1038	0.2216	0.0321	0.0950
**B**	0.3619	0.7515	-0.2297	-0.1275	-0.0413	0.0317	0.0333	0.0369	0.1200	0.1759
**Al**	-0.8708	-0.2127	0.1155	-0.1612	0.0519	-0.0144	0.1097	0.1641	0.0519	0.0458
**V**	-0.8005	-0.1503	0.1020	0.0648	0.2177	0.1153	-0.0342	0.1379	0.1080	0.0172
**Cr**	-0.0535	0.3649	0.1768	0.1359	0.3488	0.4576	-0.4963	-0.1106	0.2670	-0.0478
**Mn**	0.1774	0.7335	0.0106	-0.0916	-0.0845	0.0666	0.2234	0.1039	0.1035	0.2503
**Fe**	-0.4471	0.0997	0.0897	0.4206	-0.2179	-0.0203	0.0725	-0.5397	-0.0335	-0.2655
**Co**	-0.5809	0.1816	0.1165	0.0004	0.2658	0.3101	0.2665	0.0956	-0.0869	-0.0607
**Ni**	-0.0277	0.1304	0.3675	0.2340	-0.1043	0.2963	-0.1542	0.2095	-0.7531	0.0995
**Cu**	-0.1278	-0.0683	-0.0209	-0.3082	-0.5891	0.3952	-0.0214	0.0848	0.1662	0.1048
**Zn**	0.1197	0.5354	0.2858	-0.3574	-0.2322	0.0667	0.1609	0.0371	-0.1301	-0.2901
**As**	-0.6437	0.1171	-0.5586	0.0107	-0.0238	-0.0725	0.0060	0.0181	-0.1668	-0.0591
**Se**	0.0308	0.1378	-0.3000	0.3095	0.0369	0.4988	0.3804	0.1484	0.1462	-0.4031
**Sr**	-0.0819	0.5304	0.2411	0.1152	-0.0704	-0.3952	0.2962	0.1610	-0.0212	-0.1274
**Mo**	-0.5427	0.3407	-0.4542	0.1375	-0.0435	-0.2529	-0.0398	-0.1466	-0.1381	-0.1086
**Cd**	-0.0121	0.0173	-0.0743	-0.0844	0.2827	0.2719	0.5011	-0.5411	-0.1197	0.3769
**Sn**	0.0731	0.3024	-0.1486	-0.4801	0.3228	0.0999	-0.3490	-0.2147	-0.2221	-0.0861
**Sb**	-0.3049	0.2536	-0.7156	-0.3342	-0.1267	-0.0345	-0.1057	0.0320	-0.0781	0.1197
**Te**	0.0170	-0.4857	-0.2212	0.3632	-0.4147	0.1786	0.0387	-0.0399	-0.0408	0.2683
**Ba**	-0.3554	0.5434	0.3595	0.3298	-0.0444	-0.0455	-0.0171	0.0572	0.0894	0.3048
**Hg**	0.0412	0.2232	-0.4941	0.5130	0.0032	0.1581	-0.1896	0.2529	-0.0020	0.0411
**Tl**	-0.6232	0.3148	0.2447	0.2550	-0.1173	-0.1835	-0.2507	-0.1395	0.1426	0.1411
**Pb**	-0.4099	0.0768	0.1957	-0.3671	-0.5168	0.2066	-0.1547	-0.2015	0.0458	-0.1214
Eigenvalues	4.14	2.98	2.08	1.88	1.40	1.27	1.19	1.01	0.90	0.85
Total variance (%)	17.98	12.97	9.03	8.16	6.11	5.51	5.18	4.40	3.93	3.68
Cumulative variance (%)	17.98	30.95	39.98	48.14	54.25	59.76	64.94	69.34	73.27	76.95

Table S9. Principal component analysis of elements in wines from Fruška Gora and imported wines. Loadings of the variables for the first ten principal components, based on correlations.

	**Factor 1**	**Factor 2**	**Factor 3**	**Factor 4**	**Factor 5**	**Factor 6**	**Factor 7**	**Factor 8**	**Factor 9**	**Factor 10**
**Be**	-0.7108	0.0201	0.3271	-0.3269	0.0193	0.2047	0.1895	0.0286	-0.0795	0.2420
**B**	0.5010	-0.6539	-0.2420	-0.1426	-0.1260	-0.0191	0.0378	0.0228	-0.1322	-0.0634
**Al**	-0.8452	-0.0475	0.2944	-0.0661	0.0142	0.1560	0.1521	0.0154	-0.1602	0.1517
**V**	-0.4836	-0.0106	-0.5649	0.0846	0.3763	-0.1779	-0.2565	-0.0244	0.0636	0.1323
**Cr**	0.1110	-0.5846	-0.0421	0.1241	0.0846	0.2250	-0.0779	0.3911	0.1732	0.1226
**Mn**	0.3048	-0.7241	0.0389	0.0114	-0.1642	-0.0228	-0.0574	-0.0529	-0.1479	-0.1385
**Fe**	-0.4283	-0.0988	0.0892	0.4683	-0.1343	-0.0093	-0.0052	-0.2380	0.2305	-0.4008
**Co**	-0.4114	-0.4497	0.0973	-0.0291	0.0557	0.4193	-0.2512	-0.0622	-0.3224	0.0030
**Ni**	0.0214	-0.2310	0.1991	0.3708	0.1095	-0.0660	-0.4174	0.2198	0.1106	0.4156
**Cu**	-0.1790	0.0142	0.2320	-0.1828	-0.3890	-0.4785	-0.2788	0.0429	-0.1504	0.1658
**Zn**	0.2129	-0.4668	0.2060	-0.2067	-0.0202	-0.3120	-0.2407	-0.3752	-0.0286	0.0199
**As**	-0.5794	-0.1679	-0.5260	-0.1989	-0.0328	-0.0015	-0.0609	-0.0098	-0.0740	-0.0786
**Se**	0.0024	0.0404	-0.2001	0.2098	-0.5855	0.3021	-0.1121	-0.2549	-0.0557	0.3798
**Sr**	0.0672	-0.1780	-0.0697	0.2549	0.1116	-0.2290	0.4981	-0.5370	-0.0245	0.3909
**Mo**	-0.4646	-0.1739	-0.7272	0.0202	0.2177	-0.2257	-0.1069	-0.1059	0.1079	-0.0028
**Cd**	-0.0331	-0.0118	0.0252	-0.1184	-0.1372	0.4834	-0.3256	-0.4554	0.3452	-0.1338
**Sn**	0.1204	-0.2353	-0.0152	-0.4922	-0.0259	0.0667	0.1258	0.0866	0.6526	0.2588
**Sb**	-0.2452	-0.2840	-0.3697	-0.5242	-0.4155	-0.0417	0.1927	0.1389	-0.0655	-0.1441
**Te**	-0.2122	0.5104	-0.0769	0.3437	-0.3925	-0.1169	-0.1507	0.1436	0.0473	-0.0573
**Ba**	-0.1125	-0.7311	0.0934	0.3993	0.1100	0.0108	0.0879	0.0629	-0.0822	-0.0172
**Hg**	0.0657	-0.0594	-0.4591	0.3808	-0.4876	0.0687	0.2101	0.1806	0.0399	0.1390
**Tl**	-0.4581	-0.3858	0.2897	0.3416	-0.0594	-0.0618	0.2760	0.1019	0.2413	-0.1924
**Pb**	-0.4385	-0.2442	0.3980	-0.1175	-0.2645	-0.4192	-0.1023	-0.0019	0.2090	-0.0330
Eigenvalues	3.34	3.01	2.18	1.82	1.45	1.29	1.10	1.08	0.98	0.97
Total variance (%)	14.51	13.11	9.49	7.92	6.31	5.60	4.80	4.68	4.26	4.21
Cumulative variance (%)	14.51	27.61	37.11	45.03	51.33	56.93	61.74	66.41	70.68	74.89

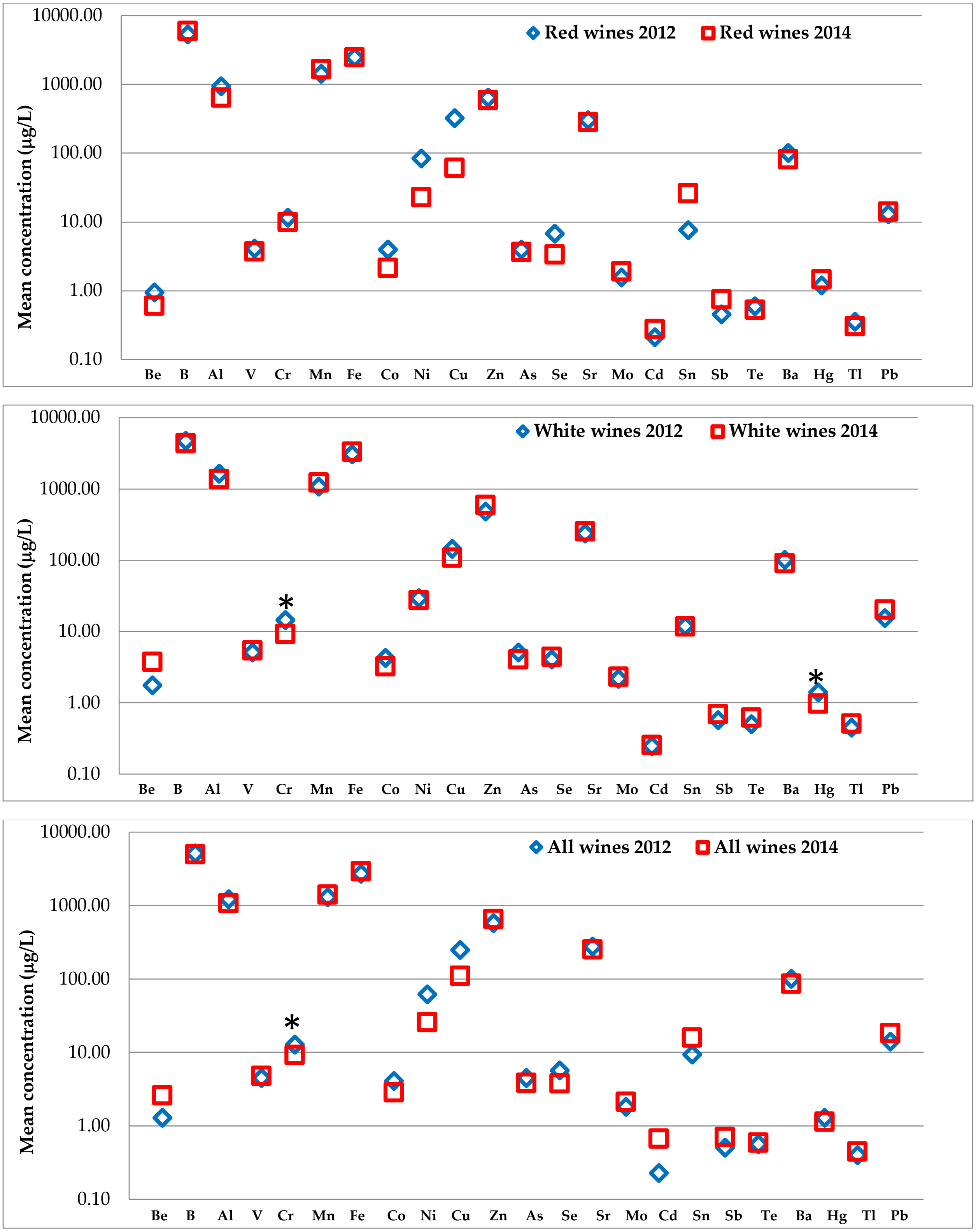

Figure S1. Comparison of mean concentrations of elements in wines from production years 2012 and 2014 (extremely hot and extremely rainy year, respectively) (* $p<0.05$).
